# MRI Assessment of Cesarean Scar Pregnancies: A Case Series

**DOI:** 10.3390/jcm12237241

**Published:** 2023-11-22

**Authors:** Rosita Comune, Carlo Liguori, Stefania Tamburrini, Francesco Arienzo, Luigi Gallo, Federica Dell’Aversana, Filomena Pezzullo, Fabio Tamburro, Pietro Affinito, Mariano Scaglione

**Affiliations:** 1 Division of Radiology, Università degli Studi della Campania Luigi Vanvitelli, 80138 Naples, Italyfe.dellaversana@gmail.com (F.D.); 2 Department of Radiology, Ospedale del Mare-ASL NA1 Centro, 80147 Naples, Italy; carlo.liguori@gmail.com (C.L.); tamburrinistefania@gmail.com (S.T.); francescoarienzo@virgilio.it (F.A.); filenapezzullo@hotmail.it (F.P.); fabio.tamburro@aslnapoli1centro.it (F.T.); 3 Department of Gynecology, Ospedale del Mare-ASL NA1 Centro, 80147 Naples, Italy; pietro.affinito@aslnapoli1centro.it; 4 Department of Medicine, Surgery and Pharmacy, University of Sassari, 07100 Sassari, Italy; 5 Department of Radiology, James Cook University Hospital, Marton Road, Middlesbrough TS4 3BW, UK

**Keywords:** cesarean scar pregnancy, embolization, MRI in pregnancy, ultrasound, diagnosis, MRI findings, transvaginal ultrasound, MRI, ectopic pregnancy

## Abstract

Cesarean scar pregnancies (CSPs) are a type of ectopic pregnancy that occur when an embryo implants within the fibrous scar tissue of a previous cesarean surgery. If not promptly detected and treated, CSPs can result in serious maternal complications, such as uterine rupture and hemorrhage. TVUS (transvaginal ultrasonography) represents the imaging of choice for the diagnosis of CSPs; however, recent studies proposed a complementary role of MRI due to its capability in soft tissue characterization that may impact the therapeutical decision-making process. The purpose of our study was to explore the role of MRI in the diagnosis and its impact on therapeutical options in CSPs. Our experience showed that MRI has a complementary role to TVSU in correctly diagnosing CSPs, identifying the type, and guiding the therapeutical approach.

## 1. Introduction

Cesarean scar pregnancy (CSP) is a type of non-tubal ectopic pregnancy that occurs when the gestational sac implants into a fibrous scar tissue of previous cesarean surgery (CS) [1]. A uterine scar dehiscence after a cesarean section is known as a ‘cesarean scar defect’, ‘niche’, ‘pouch’, or ‘isthmocele’, and it was first described by Hugh Morris in 1995 as a defect on the anterior wall of the uterine isthmus located at the site of a previous CS. Only 19 cases of CSP have been globally reported since 2002, although lately there has been a drastic increase in cesarean section rates worldwide [2], and approximately 6% of ectopic pregnancies in females with at least one previous cesarean section (CS) are expected to be CSP [1]. Its prevalence has been reported as 1/2200–1/1800 pregnancies.

The treatment of CSP represents a diagnostic dilemma, and diagnosis of CSP is generally made in the late first trimester when complications usually occur [3]. CSP can be a fatal event because of a lack of clinical signs in the early stages, and patients can directly present in emergency settings for uterine rupture and massive hemorrhage [4]. For these reasons, some authors invite practitioners to consider a CSP in every woman who presents in the first trimester to the obstetrics/gynecology practitioner with a positive pregnancy test and a history of a previous CS until proven otherwise [1].

The specific etiology of CSPs remains unclear, although inadequate healing of the uterus may determine the thinning of the muscle layer that is present in up to 60% of patients with CSPs [5,6,7,8]. Transvaginal ultrasound examination (TVUS) is currently the primary imaging modality, thanks to its ability to identify the embryo implantation site following the blood supply to the pregnant decidua from the uterine scar, although a correct and timely determination can be difficult. Determination of the exact location of the gestational sac (GS) and invasion of the placenta is necessary to estimate the patient’s bleeding risk and to propose to the patient whether to terminate or continue the pregnancy [9]. MRI has been proposed and has useful second-level imaging when ultrasound findings are equivocal and aid in confirming the diagnosis, assessing the bleeding risk, and determining the fat planes and bladder invasion before treatment [5,10,11,12,13]. Surgical, medical, and minimally invasive therapies have been described for cesarean scar pregnancy management, but therapeutical options vary between institutions and countries [14].

Particularly, the diagnostic dilemma in CSP is to distinguish the invasive form from the superficially implanted type, in order to define a correct management strategy. The purpose of our study was to retrospectively examine patients in emergency settings at our institution with an ultrasound diagnosis of CSP who underwent MRI, to assess the diagnostic accuracy of MRI and its role in patient management.

## 2. Materials and Methods

### 2.1. Patients 

From May 2021 to May 2023, patients who were referred to our institution in ER settings with a diagnosis of CSP were included in the study. Medical and surgical records and TVUS and MRI findings were reviewed. Inclusion criteria were stable patients with a suspected or confirmed diagnosis of CSP at TVUS performed during the first trimester, who underwent MRI to confirm diagnosis and classify the type of CSP, in order to choose the better management strategy. Informed consent was waived because of the observational retrospective nature of the study, and the analysis used anonymous clinical data. 

### 2.2. Transvaginal Ultrasonography

The transvaginal ultrasonographic examination was performed using a 5 MHz transvaginal transducer. The TVUS diagnostic criteria used to reach a suspected or confirmed diagnosis of CSP were the following: (i) Empty uterine cavity with a trophoblast located between the bladder and the anterior uterine wall at the presumed site of the cesarean section scar; (ii) gestational sac that is ovoid and regularly shaped, rather than distorted and collapsed as can be seen in miscarriages; and (iii) thin (1–4 mm) or discontinuous or absent myometrium between the gestational sac and the urinary bladder wall on sagittal images of the uterus through the amniotic canal sac. Additional ultrasound findings, such as (iv) vascularity of the sac on color Doppler interrogation, which can help distinguish a CSP from the avascular sac of a terminated pregnancy; (v) presence of empty endocervical canal; and (vi) negative “sliding organ sign” when gentle pressure is applied to a seen sac at the level of the internal orifice of the uterus using the endovaginal probe, can help differentiate a CSP from an ongoing spontaneous abortion [15].

### 2.3. MRI 

A multichannel receive-only surface coil was used to conduct MRI exams on a 1.5 T scanner (Amira, Siemens Medical Solutions^®^, Erlangen, Germany). The MRI protocol provides T1-weighted spin-echo (SE) pulse sequences in axial and sagittal planes, and T2 half Fourier acquisition single-shot turbo spin-echo (HASTE) in axial and sagittal planes. 

All TVUS diagnostic criteria for CSP were translated to MRI examination; additional MRI examinations were conducted at fat planes and cleavage points between the uterus and bladder, and myometrium thickness and localization of the GS were assessed [13]. 

At MRI, the cesarean scar appears sagittal T2-weighted as a hypointense area at the site of the cesarean scar with a depth of at least 1 mm; the niche was measured by the depth (the vertical distance between the base and apex of the defect), by the width (the distance of the base of the defect), and the remaining myometrium (the distance from the serosal surface of the uterus to the apex of the niche). The total myometrial thickness adjacent to the niche was measured next to the base of the defect (T) [2]. 

## 3. Results

A total of seven patients (aged between 28 and 43 years old) were included in the study. All patients were referred with a history of acute lower abdominal pain; two patients also presented metrorrhagia. All patients underwent laboratory test examinations that showed high levels of β-HCG and were first referred to the gynecological department to perform TVUS. At TVUS, a suspected diagnosis of CSP was formulated in five patients. In the others, two cases of ‘low-implanted pregnancy’ and one of pregnancy located near the CS were diagnosed. All patients were clinically and hemodynamically stable. They were admitted to the hospital and an MRI examination was performed within 48 h. Table 1 resumes the demographic and pregnancy history of patients.

MRI confirmed the CSP diagnosis in four out of seven patients. In two patients, a diagnosis of a ‘low-implanted pregnancy’, a pregnancy located near the CS scar, was formulated. In one patient, a diagnosis of a cervico-isthmic pregnancy was reached (Table 1). 

CSP patients were also classified as a Type I or II of CSP following the classification proposed by Vial et al. [9]: (i) Type I, in which the trophoblast implants on a previous cesarean scar and grows towards the uterine cavity; and (ii) Type II, in which the trophoblast implants deeply in the scar defect and progresses towards the bladder and abdominal cavity.

Particularly, among CSPs, the MRI findings of three patients were consistent with the diagnosis of Type I CSP, while the other patient was diagnosed with Type II CSP. US and MRI findings were compared in all patients (Table 2 and Table 3). In all our cases, patients underwent uterine artery embolization (UAE). Accessed from the right femoral artery, the uterine arteries were selectively catheterized using an RUC catheter and a Proareat microcatheter. A bilateral or monolateral embolization with two coils of Nester 0.35 was performed. After UAE, patients underwent hysterosuction with cannula No. 8 under ultrasound guidance and Methergin intramuscularly administered for uterotonic purposes. In two patients, minimal, ongoing bleeding occurred, and it was controlled with a bilateral ligation of the cervical branch of the uterine arteries.

## 4. Cases Presentation

### 4.1. Typical Cases

#### 4.1.1. Case 1 (CSP Type I)

**Figure 1:**
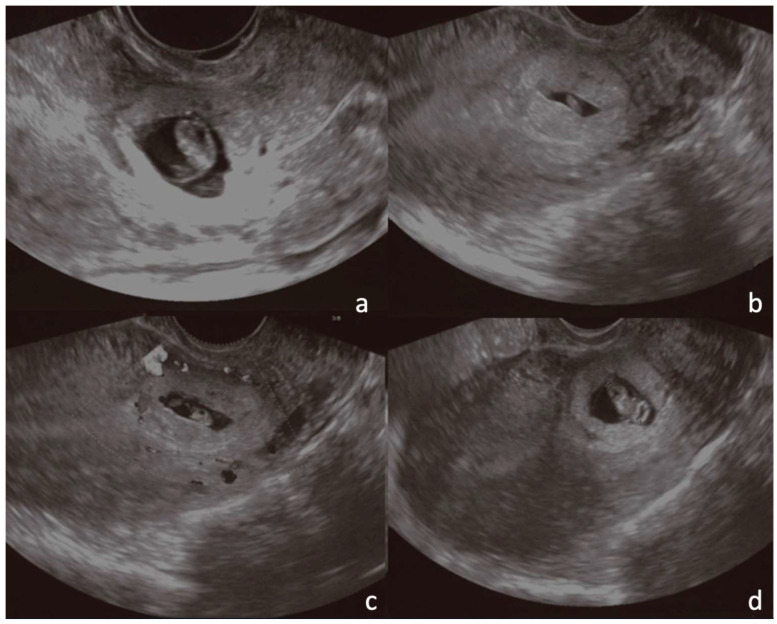
In a 30-year-old woman with a previous history of cesarean section, a cesarean scar pregnancy was detected at the 8th week of gestation. (**a**–**d**).

**Figure 2:**
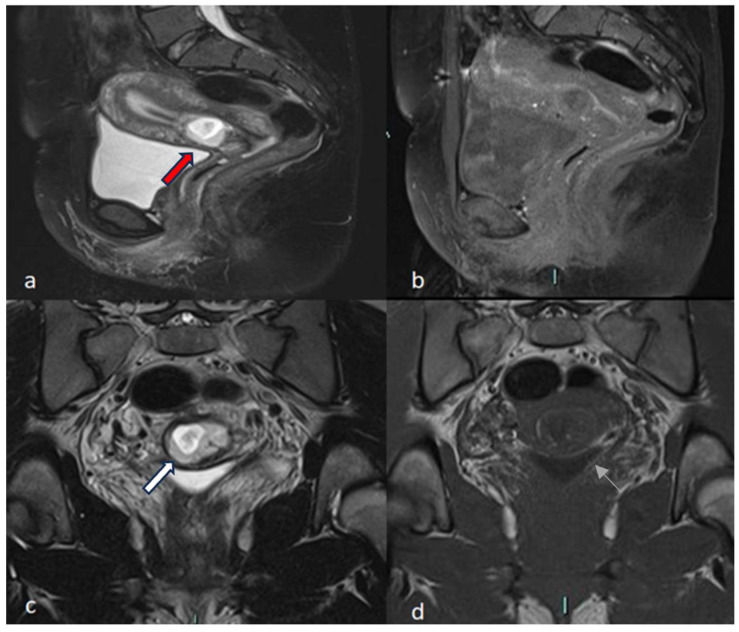
CSP Type I. Sagittal T2-weighted and fat-saturation (**a**,**b**) and axial T2-weighted and T1-weighted (**c**,**d**) MRI sequences of the pelvis showed anterior fetal pole with implant in the scar of the previous cesarean section protruding into the bladder (red arrow). A clear cleavage point with the posterior wall of the bladder was not visible. The sac was covered by a thin, hypointense layer of serosa (white arrow), and a focal interruption in the uterus serosa at anterolateral location (thin white arrow) was seen.

**Figure 3:**
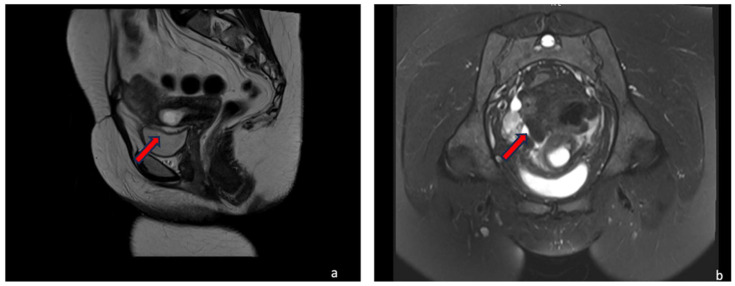
CSP Type I. Sagittal T2-weighted (**a**) and Axial (**b**) T2-weighted fat-saturation MRI sequences of the pelvis showed the gestational sac (red arrows) implanted in the lower anterior part of the uterine wall without any signs of interruption of serosa. A clear cleavage plane between the uterus and bladder is also shown.

**Figure 4:**
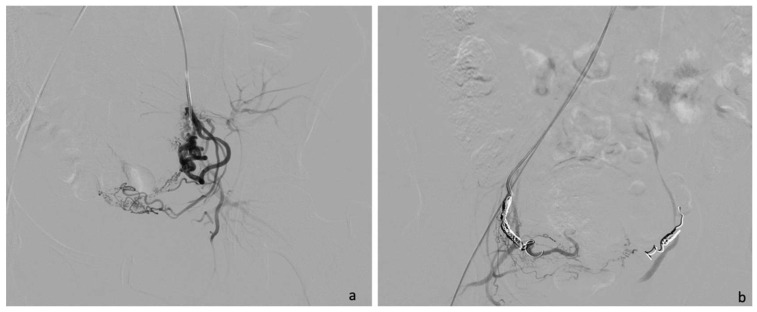
Angiographic embolization of the left uterine artery (**a**). The final control (**b**) showed no flow in both arteries, confirming the devascularization of the affected area.

A 30-year-old multiparous woman (five newborn) was referred to our institution for lower abdominal pain in the ER setting. She underwent three cesarean sections in the last three years. She underwent a TVUS examination that demonstrated an anteflexed uterus increased in size. The uterus and the cervical canal were empty. The hysterotomy scar from one of the previous cesarean sections appeared as a hypo-echoic line in the anterior lower wall. The gestational sac was visible at the isthmus within the anterior lower segment of the uterus embedded in the cesarean scar; the sac contained an 18 mm embryo without a heartbeat, indicating a pregnancy of about 8 weeks and 4 days. The myometrium layer was extremely thinned, less than 4 mm between the gestational sac and the bladder, and the trophoblast seemed to occupy the entire thickness of the myometrium (Figure 1a–d). The patient was hemodynamically stable and MRI was performed to determine the risk of an ingrown trophoblast. During the MRI examination, the patient was positioned on the left side position for her comfort. The MRI showed a gravid uterus with a placental implant at the isthmus, where the previous cesarean section scar was located (from 1 to 9 o’clock). MRI confirmed that the placenta invaded the myometrium at whole thickness, reaching the serosa but without any signs of interruption. The uterus-vesical cleavage was normal, but the placental sac clogged the cervical canal caudally by more than 50%. In T2-weighted MRI images, measurements of the niche and myometrial thickness were performed and expressed in millimeters: depth of scar, 13 mm (d); width of scar, 25 mm (w); scar myometrial thickness, <1 mm (t); and adjacent myometrial thickness, 9 mm (T) (Table 2, patient number 1; Table 3, patient number 1). The uterine cavity appeared to be modestly distended by fluid-corpuscular content, and there was no ectopic blood effusion. There was a modest amount of fluid in the Douglas pouch (Figure 2a–d). MRI confirmed the diagnosis of CSP Type I (Figure 3a,b). The patient underwent uterine artery embolization (UAE). The right femoral artery was accessed and both uterine arteries were selectively catheterized using an RUC catheter and Proareat microcatheter. Embolization was carried out using two Interlock 6 and 8 mm on the right, and two Nester 10 and 8 mm on the left. The final control angiography showed no flow in the arteries (Figure 4a,b). Subsequently, the patient underwent hysterosuction with a cannula n°8 under ultrasound guidance and intramuscularly administered Methergin for uterotonic purposes. The abortifacient material was sent to the laboratory for histological examination. No complications occurred during the treatment, and the histopathology report confirmed the diagnosis. The patient had an uneventful postoperative period and was discharged from the hospital on the fourth day.

#### 4.1.2. Case 2 (CSP Type II)

**Figure 5:**
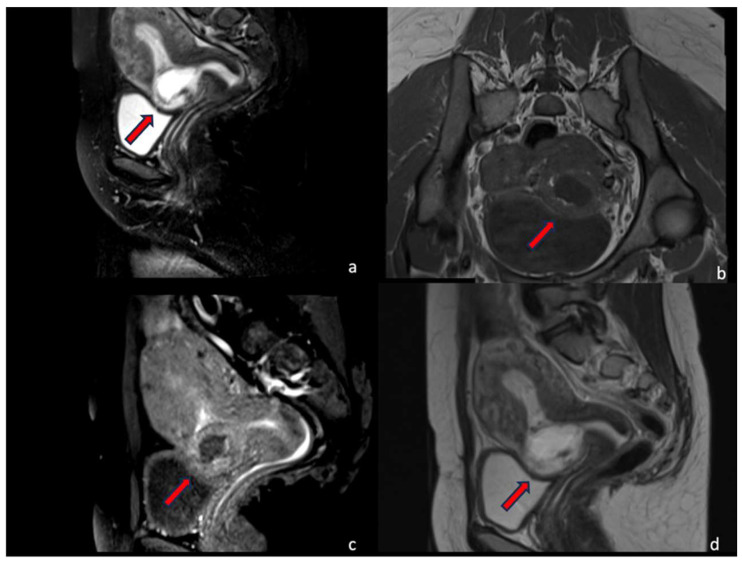
CSP Type II. Sagittal T2-weighted fat-saturation, (**a**) axial T1-weighted, (**b**) sagittal T1-weighted fat-saturation, and (**c**) sagittal T2-weighted (**d**) MRI sequences of the pelvis showed a gravid uterus with placental implant at the isthmus (red arrows) in the previous cesarean section scar. Placenta invaded the entire thickness of the myometrium and reached the serosa without any signs of interruption. A clear cleavage point with the posterior wall of the bladder was not visible.

A 35-year-old woman in her fourth pregnancy (including two cesarean deliveries and a miscarriage) was admitted to our emergency department for severe abdominal pain. Beta-hCG levels at the time of presentation were 61601 UI/L (normal value at 7 weeks of gestation 100–5000 U/L). At the TVUS, the uterus appeared lined with an empty uterine cavity at the fundic level, while there was evidence of a gestational chamber of 37.5 × 24 mm at the isthmic level, with a thin layer of myometrium, less than 4 mm between the gestational sac and the bladder. The trophoblast seemed to occupy the entire thickness of the myometrium. The gestational sac was consistent for a pregnancy of approximately 6 weeks and 6 days. A condition of scarred pregnancy was suspected; therefore, an MRI was requested to determine the risk of an ingrown trophoblast and the possible involvement of the adipose layers between the uterus and bladder. MRI showed the implantation of the placental sac at the anterior wall of the uterine body, the site of a previous cesarean section, with a full-thickness invasion of the uterine wall. The placenta reached the uterine serosa, deforming and focally interrupting it anterolaterally (Figure 5a–d). The sac, at the point of myometrium interruption, did not present a clear cleavage point with the posterior wall of the bladder, which in turn appears imprinted. The placental sac obstructed the cervical canal caudally by more than 50%. In T2-weighted MRI images, measurements of the niche and myometrial thickness were performed and expressed in millimeters: depth of scar, 29 mm (d); width of scar, 38 mm (w); scar myometrial thickness, <1 mm (t); and adjacent myometrial thickness, 18 mm (T) (Table 2, patient number 2; Table 3, patient number 2). The MRI also showed the presence of blood in the cervix and vagina. An MRI diagnosis of CSP Type II was formulated, confirming the TVUS diagnosis. Hence, uterine artery embolization and abortion were performed. Accessed from the right femoral artery, the uterine arteries were selectively catheterized using an RUC catheter and a Proareat microcatheter. A bilateral embolization with two coils of Nester0.35 was performed. A final follow-up angiography showed no flow in the arteries. The patient subsequently underwent hysterosuction with cannula No. 8 under ultrasound guidance, and Methergin was administered intramuscularly for uterotonic purposes. The minimal ongoing bleeding was controlled with a bilateral ligation of the cervical branch of the uterine arteries. (Figure 4a,b) The abortion material was sent to the laboratory for histological examination and confirmed the diagnosis of CSP. The patient was discharged on the seventh day.

#### 4.1.3. Case 3 (Low-Implanted Pregnancy) 

**Figure 6:**
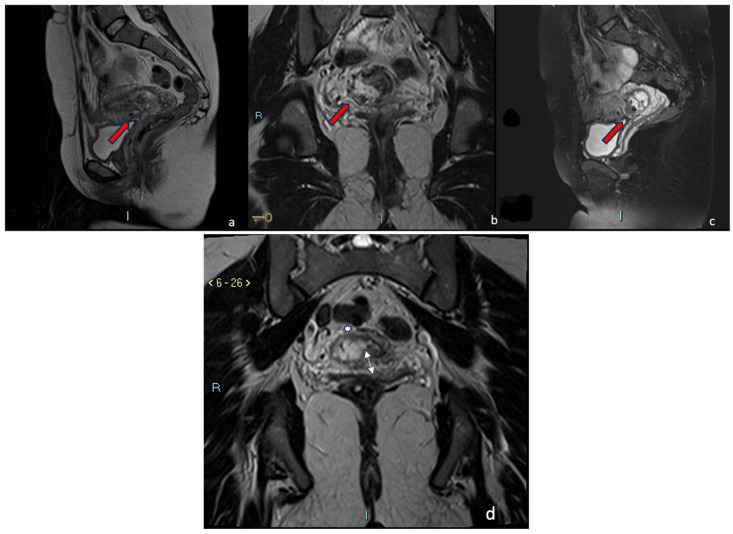
Low Implanted. Sagittal (**a**,**c**) T2-weighted and coronal T2-weighted fat-saturation (**b**) MRI sequences of the pelvis showed the gestational chamber implanted at the passage between the body and the neck of the uterus, near the previous cesarean section scar (red arrow). The serosa was focally interrupted throughout the myometrial wall, which was completely infiltrated. The posterior bladder wall did not show any evidence of invasion. (**d**) T2-weighted acquisition perpendicular to uterine cavity demonstrates implantation of the sac and internal uterine ostium with interruption of the rue internal uterine canal (white arrow) and the invasion of the myometrium (white star).

A 31-year-old woman, who had had twins with a cesarean delivery and was in her second pregnancy, was referred to our institution in an ER setting with significant blood loss (hemoglobin: 10 g/dL, normal value 11.5–17.5 g/dL). Three days before, an abortion diagnosis was made at another institution where she underwent hysterosuction and Methergin administration, then she was discharged. Beta human chorionic gonadotropin (β-hCG) levels were 9215 UI/L (the normal value at 7 weeks of gestation is 100–5000 U/L). TVUS showed an enlarged uterus and a gestational sac facing the scar of previous cesarean sections in the isthmic region. The myometrium layer was extremely thinned, with less than 4 mm between the gestational sac and the bladder, and the trophoblast seemed to occupy the entire thickness of the myometrium. The gestational chamber, which measured roughly 2.5 × 2 cm, was consistent for a pregnancy of approximately 7 weeks. The embryo had a weak and irregular heartbeat. The patient was hemodynamically stable and underwent medical therapy with ferric carboxymaltose (Ferinject 50 mg iron/mL solution for injection/infusion). She underwent MRI for diagnostic confirmation and to evaluate the cleavage point between the uterus and bladder. MRI confirmed the presence of the gestational chamber located at the passage between the body and the neck of the uterus at the right wall (from 6 to 11 o’clock). It was situated not far from the previous cesarean section scar. The serosa was focally interrupted throughout the myometrial wall, which was completely infiltrated (Figure 6a–d). The posterior bladder wall did not show any evidence of invasion. In T2-weighted MRI images, measurements of the niche and myometrial thickness were performed and expressed in millimeters: depth of scar, 12 mm (d); width of scar, 23 mm(w); scar myometrial thickness, <1 mm (t); and adjacent myometrial thickness, 8 mm (T) (Table 2, patient number 4; Table 3, patient number 4). A TVUS and MRI diagnosis of a low implanted pregnancy was formulated. Firstly, a uterine artery embolization and an abortion were carried out. Particularly, using an RUC catheter and a Proareat microcatheter, the uterine arteries were selectively catheterized after being accessed from the right femoral artery, and a bilateral embolization using two coils of Nester 0.35 was carried out. A final follow-up angiography revealed no artery flow. US-guided hysterosuction was carried out. The diagnosis was supported by a histological examination. The patient was released on the third day after an ultrasound check.

## 5. Discussion

MRI is a multiparametric imaging modality that, unlike TVUS, permits the practitioner to investigate soft tissues with high sensitivity. MRI can be used also to guide the management of CSP. Cesarean scar pregnancies, which were once considered rare, are becoming more common due to the increase in elective cesarean surgeries and improved diagnostic capabilities with transvaginal ultrasound. Although the exact pathogenesis is still not fully understood, the presence of deep chorionic villi within the uterine wall as a result of the dehiscence of a scar likely results in a further invasion of the extravillous trophoblast into the uterine wall, giving it greater access to the deep myometrium. The anterior lower uterine region is believed to have poor vascularity, which may impede healing after a cesarean surgery in some women, making this area vulnerable to small dehiscent tracts or defects where the trophoblast can implant. [7,8,16]. CPS may present with various symptoms which can range from vaginal bleeding to lower abdominal pain. However, several patients can be asymptomatic or only mildly symptomatic, and become aware of a CSP only when detected by TVUS. Different types of CSP have been described: CSP with progression to the cervico-isthmic space or uterine cavity (endogenic) or with deep invasion of the scar defect with progression toward the bladder and abdominal cavity (exogenic). The endogenic type of CSP could result in a viable pregnancy, yet with a high risk of bleeding at the placental site. The exogenic type could be complicated with uterine rupture and bleeding early in pregnancy [1] Vial et al. proposed two types of cesarean scar pregnancies (CSPs): (i) Type 1, in which the trophoblast implants on a previous cesarean scar and grows towards the uterine cavity; and (ii) Type 2, in which the trophoblast implants deeply in the scar defect and progresses towards the bladder and abdominal cavity [15]. The correct identification of each type is important due to the different prognoses. Particularly, Type 1 can result in a viable pregnancy with a high risk of bleeding, while Type 2 is more likely to rupture. A pregnancy that is located near the CS scar should be called a ‘low-implanted pregnancy’ and not a CSP (94% agreement), so a low-implanted pregnancy is defined as any pregnancy implanted near the niche/CS scar without being in direct contact with it [9]. A prompt early diagnosis and correct classification are necessary to avoid maternal complications and improve the patient’s prognosis. The main reported treatments for CSP include drug or surgical termination or a minimally invasive UAE combined with medical therapy of the pregnancy at early stages, in order to reduce the uterine rupture risk and severe bleeding which may be caused by the uterus invasion of the placenta [14,17,18,19]. However, it has been reported that, especially at earlier stages, CSP may be only mildly symptomatic, with no typical clinical findings, resulting in a late diagnosis and treatment and more severe outcomes [20]. Thus, to avoid a delayed or missed diagnosis and to improve diagnostic efficiency, TVUS is usually performed at early pregnancy stages in patients with an history of previous uterine surgery or cesarean sections [21,22]. The sensitivity of TVUS for diagnosing cesarean scar pregnancies is reported to be 84.6% [23], and it is still the preferred imaging method. It has been reported that the optimal gestational age to carry out the ultrasound for the evaluation of a CSP is 6–7 weeks (88–94% agreement) [23]. TVUS is currently the gold standard mainly due to ultrasound criteria that allow for an accurate diagnosis [15]. However, TVUS still has some limitations; US still remains an operator-dependent technique, and its sensitivity may be lower in case of low operator expertise. Moreover, TVUS does not offer a panoramic view of the pelvic area, being unable to evaluate the eventual invasion of adjacent organs such as the bladder. For these reasons, some studies reported a non-negligible rate of misdiagnosis and missed diagnosis of CSP at the US examination [24]. As a safe imaging technique during pregnancy, not requiring contrast agents and being only mildly affected by the patient’s physical constitution and other conditions, MRI is considered as a highly effective, complementary, and safe tool in gestational imaging. Several studies already assess the diagnostic capabilities of MRI in the diagnosis of various gestational diseases, such as ectopic pregnancies [25]. Regarding CSP, MRI may be potentially useful in cases where ultrasound gives uncertain or doubtful results. At the MRI, T1- and T2-weighted sagittal and axial sequences can reveal the exact position of the gestational sac implantation, a more precise evaluation of a cesarean scar pregnancy, as well as the involvement of surrounding organs such as the bladder (like CSP Type II) [26]. Misdiagnosis of this condition as other types of ectopic pregnancy, such as cervical pregnancy or incomplete abortion, may lead to inappropriate treatment with curettage and subsequent dangerous bleeding [16]. In our cases, MRI was performed not only to confirm the diagnosis but also to assess the growth of the embryo towards the peritoneal cavity (CSP Type II) that could not be ruled out through sonography alone. In one case, the MRI revealed an intrauterine pregnancy protruding through the myometrium of the lower uterine segment and pushing against the superior side of the urinary bladder without invading the bladder wall. In the other case, the placenta reached the uterine serosa, deforming and focally interrupting it, without presenting a clear cleavage point with the posterior wall of the bladder, which in turn appears imprinted. A diagnostic confirmation is necessary for planning the therapeutic approach and before definitive surgical intervention. With the increasing availability of minimally invasive uterine artery embolization and the superior soft tissue characterization and anatomical information provided by MRI, patients and clinicians can consider conservative care as the first option [27,28]. Conservative management can be chosen as an initial treatment option, especially when minimally invasive UAE is available, and combined with medical therapy, this may make it more feasible to preserve fertility in CSP cases. Although there is no standard approach, early intervention is necessary to reduce complications and maternal morbidity, regardless of the treatment option selected. Several authors promote a conservative mindset when using medical care. The biggest disadvantage of medical treatment is the slow resolution of the pregnancy, which increases the risk of hemorrhage and rupture, where a hysterectomy then becomes necessary [29,30]. The treatment plan should be tailored to the specific needs of each patient, requiring extensive consultation between the patient and the clinicians. Finally, the decision should be based on various factors, including the patient’s clinical presentation, gestational age, desire for future fertility, and the clinician’s experience with this condition. In all our cases, patients with a confirmed MRI diagnosis of CSP were treated with arterial embolization due to the high risk and recurrence of uterine bleeding [2,31,32]. A recent study [12] suggested that contrast-enhanced MRI can be used as a reliable adjunct and initial imaging modality for diagnosing CSP. The imaging features of contrast-enhanced MRI may result in a more accurate diagnosis before specific treatment for CSP. Hoffmann et al. evaluated the use of MRI and TVUS in the diagnosis of CSP; the authors reported that there was a significant difference between the diagnostic results of 3.0 T MRI and TVUS, with MRI showing better and more clear images of the uterine scar, resulting in an even higher accuracy in the measurement of lower uterus wall thickness [33]. In summary, TVUS remains the imaging of choice in the diagnosis of CSPs with high sensitivity and specificity, but MRI can provide additional information for differential diagnosis, depth assessment, and treatment planning in complex or equivocal cases. The choice of imaging modalities should be guided by the clinical scenario and the expertise of the healthcare team. Our experience suggests that MRI may have an even higher sensitivity than TVUS, suggesting a central role in the early diagnosis and treatment planning of CSPs. In clinical practice, MRI and TVUS may be complementary examinations. Particularly, TVUS may play a central role in the first and emergency evaluation of patients, while MRI should be performed in stable patients to confirm the TVUS diagnosis and better classify patients, with significant prognostic and therapeutical values.

## 6. Conclusions

TVUS is still the preferred imaging method for CSP diagnosis; however, MRI is becoming more important in examining CSPs due to its improved soft tissue characterization and anatomical information. MRI images may clearly demonstrate the location of the CSP gestational sac implant, the depth of invasion of the muscle layer, and the interactions between surrounding tissues and the cesarean scar. All these features suggest a potential pivotal role of MRI in the diagnosis and in the choice between the various therapeutic options. Our experience showed the potential role that MRI may have in the diagnosis and classification of CSP, being able to easily confirm uncertain diagnoses and identify the correct type of CSP in each patient. However, further studies are needed to better clarify the role of MRI in early CSP diagnoses and to evaluate a combined imaging approach in high-risk patients and in patients with doubtful TVUS findings.

## Figures and Tables

**Table 1:** Demographic and pregnancy history of patients.

Patient Number	Ages	Previous Pregnancies	Previous Cesarian Section	GA (Weeks) at TVUS Exam	Final Diagnosis
1	30	4	3	8 w and 4 d	CSP I
2	35	3	2	6 w and 6 d	CSP II
3	43	3	1	8 w and 3 d	CSP I
4	31	1	1	7 weeks	Low-implanted pregnancy
5	35	1	1	7 w and 4 d	CSP I
6	28	2	2	10 w	Low-implanted pregnancy
7	32	2	2	9 w and 5 d	Cervico-isthmic pregnancy

Abbreviations: w = weeks, d = days, CSP = cesarian scar pregnancy.

**Table 2:** Comparison of US and MRI findings.

		Empty Uterus	Empty Endocervical Canal	Trophoblast within the CS	GS Ovoid and Regularly Shaped	Vascularity of GS at CD	Myometrium (Measured between the Gestation Sac and Bladder Wall) (1–4 mm) or Discontinuous or Absent	Cleavage Points between the Uterus and Bladder	Negative “Sliding Organ Sign”	Diagnosis
Patient 1	TVUS	+	+	+	+	+	+	−	+	CSP I
	MRI	+	+	+	+	−	+	+	−	
Patient 2	TVUS	+	+	+	+	+	+	−	+	CSP II
	MRI	+	+	+	+	−	+	+	−	
Patient 3	TVUS	+	+	+	+	+	+	−	+	CSP I
	MRI	+	+	+	+	−	+	+	−	
Patient 4	TVUS	+	+	−	+	+	+	−	+	Low-implanted pregnancy
	MRI	+	+	−	+	−	+	+	−	
Patient 5	TVUS	+	+	+	+	+	+	−	+	CSP I
	MRI	+	+	+	+	−	+	+	−	
Patient 6	TVUS	+	+	−	+	+	+	−	+	Low-implanted pregnancy
	MRI	+	+	−	+	−	+	+	−	
Patient 7	TVUS	+	−	−	+	+	+	−	−	Cervico-isthmic pregnancy
	MRI	+	−	−	+	−	+	+	−	

Abbreviations: MRI = magnetic resonance imaging, TVUS = transvaginal ultrasonographic, CSP = cesarian scar pregnancy, CD = color doppler, GS = gestational sac, CS = cesarean scar.

**Table 3:** MRI measures of cesarean scar defect.

Patient Number	Depth of Scar mm (d)	Width of Scar (w)	Scar Myometrial Thickness (t)	Adjacent Myometrial Thickness (T)
#1	13 mm	25 mm	<1 mm	9 mm
#2	29 mm	38 mm	<1 mm	18 mm
#3	10 mm	26 mm	<1 mm	3 mm
#4	12 mm	23 mm	<1 mm	8 mm
#5	9 mm	18 mm	2 mm	10 mm
#6	6 mm	22 mm	<1 mm	9 mm
#7	15 mm	49 mm	<1 mm	8 mm

## Data Availability

The data presented in this study are available on request from the corresponding author. The data are not publicly available due to [R.C., M.S.].
